# Artificial Intelligence in Obstetrics and Gynecology Nursing: Clinical, Educational, and Ethical Perspectives

**DOI:** 10.7759/cureus.106026

**Published:** 2026-03-28

**Authors:** Kuppusamy Sivasankari, Santhoshkumari Mohan

**Affiliations:** 1 Obstetrics and Gynecology Nursing, Jawaharlal Institute of Postgraduate Medical Education and Research, Puducherry, IND; 2 Obstetrics and Gynecology, Indira Gandhi Medical College & Research Institute, Puducherry, IND

**Keywords:** artificial intelligence, clinical decision support, deep learning, digital health, machine learning, maternal health, nursing education, obstetrics and gynecology nursing

## Abstract

Artificial intelligence (AI) is rapidly transforming healthcare by enabling advanced data analysis, predictive modeling, and intelligent clinical decision support systems. In obstetrics and gynecology (OBG) nursing, AI technologies are increasingly recognized as valuable tools for improving maternal and women’s healthcare outcomes. These technologies facilitate early identification of high-risk pregnancies, enhance fetal monitoring accuracy, and support gynecologic cancer screening. This narrative review examines current evidence on the emerging applications of AI in obstetric and gynecologic practice, with particular emphasis on its relevance to nursing roles and responsibilities. A structured search of electronic databases, including PubMed, Scopus, Excerpta Medica Database (EMBASE), Cumulative Index to Nursing and Allied Health Literature (CINAHL), and Web of Science, was conducted for studies published between January 2010 and July 2025. The search yielded 612 records, of which 62 studies met the inclusion criteria and were included in the thematic synthesis. Key domains explored include predictive analytics for maternal risk assessment, AI-assisted clinical decision support systems for labor and emergency management, wearable and remote monitoring technologies for continuous maternal and fetal surveillance, and image-based diagnostic tools used in gynecologic oncology screening and early disease detection. The review also highlights applications of AI in nursing education, including adaptive learning platforms and simulation-based training that enhance clinical reasoning and preparedness for obstetric emergencies. Ethical and implementation challenges, including algorithmic bias, data privacy, transparency, and equitable access, are also discussed. While AI shows promising potential to improve diagnostic accuracy, support evidence-based decision-making, and optimize workflow efficiency, much of the current evidence remains in developmental or pilot phases, with limited large-scale validation. Overall, AI has the potential to strengthen obstetrics and gynecology nursing practice by facilitating proactive, data-driven care while preserving the essential human-centered and compassionate nature of nursing in maternal and women’s health.

## Introduction and background

Obstetrics and gynecology (OBG) nursing encompasses comprehensive care across the reproductive life span, including antenatal, intrapartum, postpartum, and gynecologic health services. Despite measurable global progress in maternal health, preventable morbidity and mortality remain major public health concerns. According to the World Health Organization, although the global maternal mortality ratio has declined over the past two decades, disparities persist, particularly in low- and middle-income countries [1]. In India, maternal mortality continues to exceed the Sustainable Development Goal target of 70 per 100,000 live births, highlighting ongoing gaps in timely diagnosis, quality of care, and equitable service delivery [2]. OBG nurses frequently serve as frontline providers responsible for early recognition of life-threatening complications such as preeclampsia, postpartum hemorrhage, sepsis, and fetal distress, often within resource-constrained environments.

Traditional maternal monitoring relies on intermittent vital sign assessment and manual interpretation of cardiotocography (CTG), which may be influenced by subjective judgment and are associated with interobserver variability and delayed recognition of complications [3]. Delayed diagnosis in gynecologic oncology, such as cervical cancer, remains a challenge, especially in low-resource settings; AI-assisted cytology has demonstrated improved accuracy in detecting precancerous lesions [4]. These systemic challenges underscore the need for innovative, data-driven strategies that can enhance clinical vigilance while preserving patient-centered care.

Artificial intelligence (AI) has emerged as an advanced analytical approach capable of supporting clinical decision-making through machine learning, deep learning, natural language processing, and predictive analytics [5,6]. In obstetric care, AI models have demonstrated utility in predicting gestational diabetes mellitus, preeclampsia, and preterm birth using electronic health records and biometric data [7-9]. AI-enhanced CTG interpretation systems have also shown promise in reducing interobserver variability and improving early detection of abnormal fetal heart rate patterns [10].

In gynecologic practice, AI-driven image analysis has improved diagnostic accuracy in cervical cytology and breast cancer screening, supporting earlier identification and timely referral [11,12]. For OBG nurses, who play critical roles in screening programs, patient counseling, and care coordination, such technologies may enhance efficiency, reduce cognitive burden, and strengthen clinical outcomes. Beyond direct patient care, AI applications extend into nursing education, simulation-based training, and research analytics, supporting workforce preparedness and evidence generation within nursing practice [13]. This review specifically focuses on AI applications relevant to obstetrics and gynecology nursing, emphasizing their implications for clinical practice, nursing decision-making, education, and implementation challenges.

However, the integration of AI into nursing practice raises ethical, legal, and infrastructural considerations. Concerns regarding algorithmic bias, data privacy, transparency, and equitable implementation must be addressed to ensure that technological advancement does not inadvertently widen healthcare disparities [14,15]. Successful adoption requires not only technical innovation but also digital literacy, governance frameworks, and nurse-led participation in system design and evaluation. Despite growing research on AI in healthcare, evidence focusing specifically on its application and implications for obstetrics and gynecology nursing remains fragmented.

Given the rapid evolution of AI technologies in maternal and women’s health, there is a need to synthesize evidence specifically relevant to nursing practice. This narrative review aims to examine the applications of artificial intelligence in obstetrics and gynecology nursing, with a focus on clinical decision support, maternal risk prediction, nursing education, and implementation challenges, including ethical and system-level considerations.

## Review

Study design

This narrative review was conducted to provide a comprehensive overview of current evidence regarding the applications of artificial intelligence (AI) in obstetrics and gynecology (OBG) nursing. Narrative reviews are particularly appropriate for emerging and rapidly evolving fields, where heterogeneity in study design, outcomes, and implementation contexts may limit the feasibility of quantitative synthesis [16]. This approach allows for thematic integration of diverse forms of evidence while maintaining relevance to clinical nursing practice. This review followed a structured search approach; however, it was not conducted as a formal systematic review and did not adhere to Preferred Reporting Items for Systematic Reviews and Meta-Analyses (PRISMA) guidelines. Study selection and synthesis were interpretive in nature, aimed at providing a thematic overview of the literature rather than a protocol-driven, reproducible systematic analysis.

Search strategy

A structured search of electronic databases including PubMed, Scopus, Excerpta Medica Database (EMBASE), Cumulative Index to Nursing and Allied Health Literature (CINAHL), and Web of Science was performed to identify relevant literature published between January 2010 and July 2025. Keywords and Medical Subject Headings (MeSH) terms included: “artificial intelligence,” “machine learning,” “deep learning,” “natural language processing,” “predictive analytics,” “nursing,” “midwifery,” “maternal health,” “obstetrics,” and “gynecology.” Boolean operators (AND/OR) were applied to refine the search combinations. An example search strategy used in PubMed was: (“artificial intelligence” OR “machine learning”) AND (“obstetrics” OR “gynecology” OR “maternal health”) AND (“nursing” OR “midwifery”). Only articles published in English were included. Duplicate records identified across databases were removed prior to screening. Title and abstract screening was performed independently by the authors to identify potentially relevant studies, followed by full-text review to confirm eligibility. Any discrepancies were resolved through discussion and consensus. In addition to database searching, reference lists of key articles were manually screened to identify further relevant publications. A study selection flowchart has been added to illustrate the screening and inclusion process. Reporting guidance related to AI-based clinical research was also reviewed to contextualize methodological standards in the field [17].

Eligibility criteria

Peer-reviewed original research studies, systematic reviews, methodological papers, and relevant policy or ethical analyses were considered eligible if they addressed AI applications within obstetric, gynecologic, or nursing-related contexts. Both quantitative studies (e.g., diagnostic accuracy studies, cohort studies, predictive modeling research) and qualitative or implementation-focused studies (e.g., nurse perspectives, ethical considerations) were included. Studies were included if they demonstrated clear relevance to obstetrics and gynecology nursing practice, either through direct involvement of nurses, implications for nursing decision-making, patient care, education, or workflow processes. Studies conducted in obstetric or gynecologic settings without explicit nursing outcomes were included only if their findings had clear applicability to nursing roles and responsibilities. During synthesis, different types of evidence were integrated using a thematic approach, where findings from quantitative, qualitative, and review-based studies were collectively interpreted to identify recurring themes relevant to clinical practice, education, and implementation. Studies discussing AI exclusively in general medical specialties without relevance to women’s health or nursing practice, conference abstracts without full text, and non-English publications were excluded.

Data extraction and thematic synthesis

**Figure 1:**
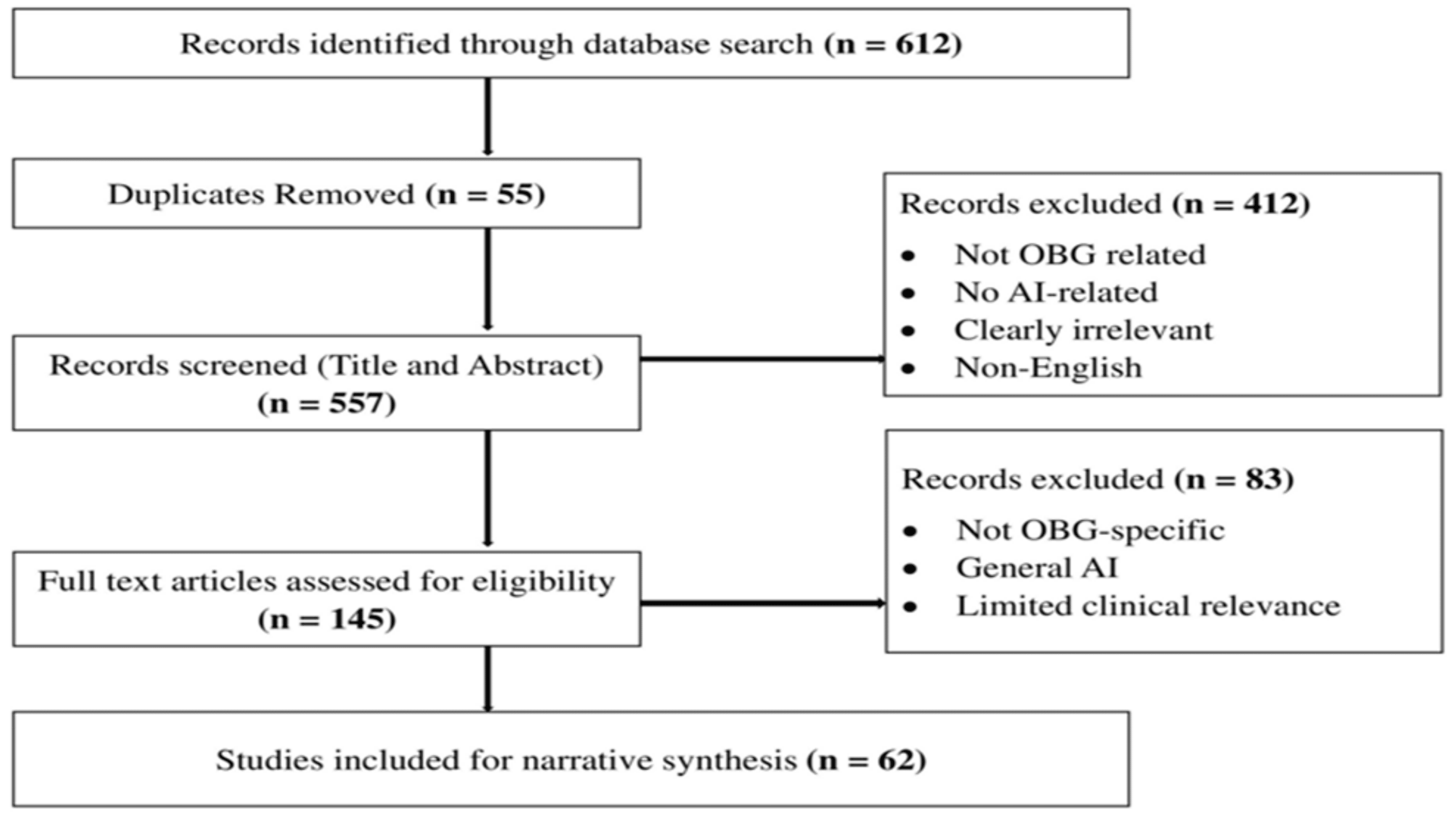
Flow of study selection and exclusion process for the narrative review OBG: obstetrics and gynecology, AI: artificial intelligence.

The initial search yielded 612 records. After removal of duplicate records, studies were screened based on title and abstract to identify potentially relevant articles. These articles were then assessed through full-text review for eligibility based on the predefined inclusion and exclusion criteria. Finally, 62 studies met the inclusion criteria and were included in the thematic synthesis. A formal quality appraisal of included studies was not performed, consistent with the narrative review approach. Data extraction was performed independently by the authors using a standardized data extraction framework. Extracted information included the type of AI application, clinical or educational domain, population or setting, relevance to nursing practice, reported outcomes, and ethical or implementation challenges. Any discrepancies in data extraction were resolved through discussion and consensus. A study selection flowchart has been added (Figure 1) to illustrate the screening and inclusion process.

Given the conceptual diversity of the included studies, a statistical meta-analysis was not performed. Instead, findings were organized into thematic domains relevant to OBG nursing practice. Themes were developed inductively based on recurring patterns identified across the included studies, rather than being predefined. These domains included predictive analytics in maternal care, clinical decision support systems, wearable and remote monitoring technologies, gynecologic oncology applications, nursing education innovations, and ethical and implementation considerations. This synthesis approach enabled identification of both current applications and evidence gaps relevant to frontline nursing practice across diverse healthcare settings. A summary of key studies describing artificial intelligence applications in obstetrics and gynecology and their implications for nursing practice is presented in Table 1.

**Table 1:** Selected studies on artificial intelligence applications in obstetrics and gynecology and their implications for nursing practice AI: artificial intelligence, DL: deep learning, ML: machine learning, FHR: fetal heart rate, GDM: gestational diabetes mellitus, PE: preeclampsia, CTG: cardiotocogram.

Study	Study Design/AI Model	Key Findings	Conclusion	Nursing Implications
Wentzensen et al., 2021 [4]	DL automation in cervical cancer screening	AI improved accuracy of cervical cytology interpretation	AI may enhance cervical cancer screening programs	Nurses may play a key role in implementing AI-assisted screening programs, particularly in supporting early detection and patient follow-up
Topol, 2019 [5]	AI-based clinical decision support systems	AI-assisted tools improved diagnostic accuracy and clinical workflow efficiency	AI has the potential to transform healthcare delivery including obstetric and gynecologic practice	AI-supported decision tools may have potential applications in supporting nursing decision-making in obstetric and gynecologic settings
Ranjbar et al., 2022 [9]	Systematic review of ML models for preeclampsia prediction	AI models demonstrated potential in predicting PE using clinical and biochemical parameters	AI may enhance early risk assessment in pregnancy	AI-based tools may support nurses in strengthening antenatal risk assessment and early identification of high-risk pregnancies
Aeberhard et al., 2024 [10]	ML analysis of cardiotocography signals	AI algorithms improved detection of abnormal FHR patterns and reduced interobserver variability	AI-assisted CTG interpretation may enhance fetal monitoring accuracy during labor	AI-assisted CTG interpretation systems may support nurses involved in intrapartum monitoring by reducing variability and aiding early detection
Hu et al., 2019 [11]	DL algorithm for cervical cytology screening	AI demonstrated high sensitivity and specificity in detecting abnormal cervical cells	AI-based screening tools may support early detection of cervical cancer	Nurses in gynecological screening programs can use AI-supported cytology tools to facilitate early detection and referral of women at risk of cervical cancer
McKinney et al., 2020 [12]	DL system for mammography screening	AI system achieved diagnostic performance comparable to expert radiologists in breast cancer detection	AI may improve accuracy and efficiency of breast cancer screening programs	Nurses working in women’s health and screening services can assist in implementing AI-supported diagnostic technologies and improving patient education regarding screening
Joshi et al., 2024 [18]	ML algorithms for gestational diabetes prediction	ML models showed improved predictive performance in identifying women at risk of GDM	AI models may support early detection and prevention strategies	Nurses can use AI-based screening results to initiate early lifestyle education, dietary counseling, and monitoring during antenatal care
Zhang et al., 2022 [19]	ML model using electronic health record data	AI predicted GDM with higher predictive accuracy	AI tools may support early screening and preventive management of gestational diabetes	Nurses can support early screening programs and provide lifestyle counseling and monitoring for women identified as high risk for gestational diabetes
Arnaout et al., 2021 [20]	DL model for prenatal ultrasound analysis	AI-assisted ultrasound improved detection of congenital heart disease in fetuses	AI can enhance prenatal diagnostic imaging and clinical decision making	Nurses involved in prenatal care can assist in coordinating early referral, counseling families, and supporting multidisciplinary care when congenital anomalies are detected.

Review

Predictive Analytics in Maternal Care

AI-driven predictive models are increasingly being applied in maternal healthcare to identify women at risk for complications such as preeclampsia, gestational diabetes mellitus (GDM), preterm birth, and postpartum hemorrhage. By analyzing large datasets derived from electronic health records, laboratory results, and sociodemographic variables, machine learning algorithms can detect complex risk patterns earlier than conventional screening approaches [21]. Machine learning models have shown potential to outperform traditional statistical methods in predicting gestational diabetes and hypertensive disorders of pregnancy; however, this evidence is not uniform and often derives from retrospective or proof-of-concept studies [7,8,17]. Many models require further external validation, calibration across diverse populations, and evaluation in real-world clinical settings before routine implementation can be recommended.

Early risk stratification may enable OBG nurses to intensify monitoring, provide targeted counseling, and facilitate timely referrals for specialist care. Emerging AI innovations, including simulation models of maternal-fetal health trajectories, offer potential for anticipatory risk assessment and personalized clinical support [17,22,23]. However, most of these tools remain in developmental or pilot phases, with limited large-scale implementation in routine practice.

These predictive approaches may be particularly valuable in resource-limited settings, where early identification of high-risk pregnancies can significantly influence maternal and neonatal outcomes. Nevertheless, considerations related to data quality, model generalizability, and integration into existing clinical workflows remain critical. Overall, AI has the potential to support proactive clinical decision-making while maintaining the nurse’s central role in assessment, communication, and compassionate care delivery.

Clinical Decision Support Systems (CDSS)

Clinical decision support systems (CDSS) integrate patient-specific data with clinical knowledge to assist healthcare providers in decision-making. While traditional CDSS are often based on rule-based algorithms and clinical guidelines, emerging systems incorporate artificial intelligence techniques to enable more adaptive and data-driven support. In intrapartum care, digital tool interpretation of cardiotocography (CTG) has shown potential to improve detection of abnormal fetal heart rate patterns and reduce interobserver variability [24]. However, it is important to note that not all CTG interpretation tools currently in use are AI-driven, and evidence varies across systems. Similarly, maternal early warning systems are widely used to support early identification of obstetric complications such as sepsis, postpartum hemorrhage, and hypertensive disorders. While some of these systems are enhanced by predictive analytics, many remain based on standardized scoring frameworks rather than true early warning system models [24,25]. These tools may support nurses by generating alerts based on physiological parameters, potentially reducing cognitive workload and promoting timely intervention. However, the extent to which these systems incorporate advanced AI techniques and their effectiveness in real-world clinical settings remains variable. Importantly, CDSS tools are intended to complement, not replace, clinical judgment. Successful implementation requires adequate training, integration into clinical workflows, and the ability to critically appraise automated recommendations to avoid overreliance. When appropriately applied, AI-supported and digital decision support systems may enhance nursing practice and contribute to improved patient safety.

Wearables and Remote Monitoring

Wearable technologies and remote monitoring platforms are increasingly being explored to support maternal and neonatal health. Devices capable of tracking physiological parameters such as blood pressure, heart rate, glucose levels, and fetal activity can enable continuous data collection outside traditional clinical settings [26]. While many wearable systems primarily function as data collection tools, integration with advanced analytics, including artificial intelligence, is an emerging area. AI-enabled platforms have the potential to analyze large volumes of continuously generated data to identify trends and predict clinical deterioration; however, such applications are still evolving and are not yet widely implemented in routine obstetric care. Wearable electrocardiogram (ECG) patches and photoplethysmography-based sensors have demonstrated potential in detecting arrhythmias and physiological abnormalities during pregnancy [27]. Similarly, remote monitoring platforms allow healthcare providers, including nurses, to oversee high-risk pregnancies outside hospital settings, particularly in rural or underserved areas, facilitating timely communication and intervention [26-28].

In neonatal care, continuous monitoring of respiratory patterns and oxygen saturation may support early recognition of clinical deterioration. Although some systems incorporate predictive analytics, many currently used technologies rely on threshold-based alerts rather than fully AI-driven models. Overall, wearable and remote monitoring technologies may enhance continuity of care and support early intervention; however, the evidence base remains heterogeneous, and further research is needed to establish the effectiveness, scalability, and integration of AI-driven approaches in real-world maternal and neonatal care settings.

Applications in Gynecologic Oncology

**Figure 2:**
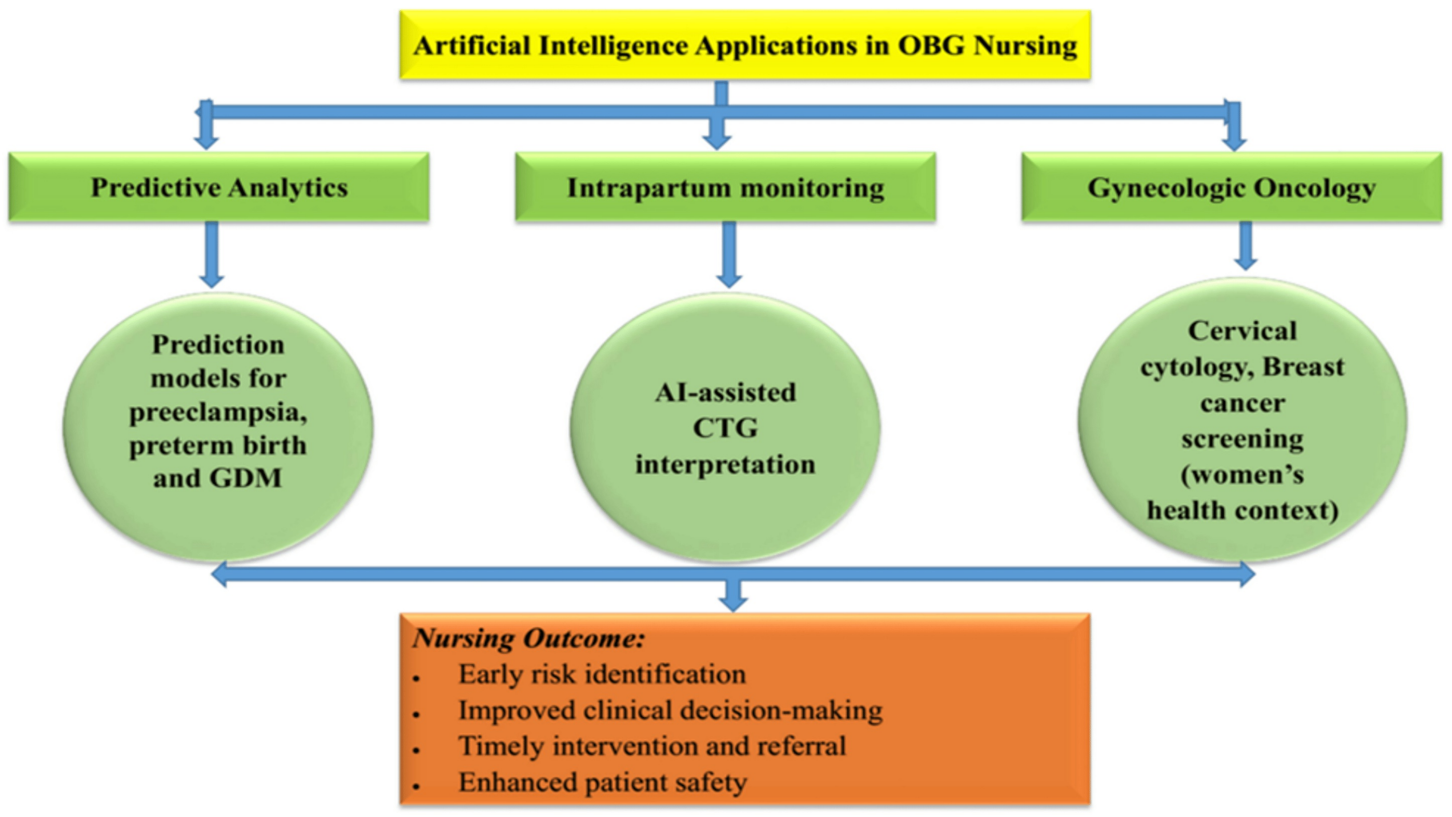
Domains of artificial intelligence applications in obstetrics and gynecology nursing, including predictive analytics, intrapartum monitoring, wearable technologies, and gynecologic oncology screening The figure was created by the authors using Microsoft PowerPoint (Microsoft Corporation, Redmond, Washington, United States) then converted to JPEG. OBG: obstetrics and gynecology, GDM: gestational diabetes mellitus, CTG: cardiotocography.

Artificial intelligence has significantly advanced cancer screening, diagnostic accuracy, and early detection within gynecologic oncology. Hou et al. [29] demonstrated that deep learning algorithms applied to cervical cytology and dual-stain testing improve sensitivity and specificity compared to conventional manual interpretation [4], while also supporting large-scale screening programs in resource-limited settings. Similarly, AI-assisted colposcopic image analysis enables earlier detection of cervical lesions, particularly where access to expert interpretation is limited [11]. In ovarian cancer care, AI-based predictive models and imaging analysis have shown potential in supporting risk stratification and early detection, although evidence remains limited and continues to evolve. In addition, AI applications in breast cancer screening, particularly in mammographic interpretation, have demonstrated performance comparable to experienced radiologists in some studies [12]. Although breast cancer is not strictly classified under gynecologic oncology, it remains a critical component of women’s health, and its inclusion here reflects the broader scope of OBG nursing responsibilities in screening and patient education. For OBG nurses, who play key roles in screening programs, patient counseling, and care coordination, these technologies may support early detection, improve workflow efficiency, and facilitate timely referral pathways [13]. By integrating AI insights with clinical expertise, nurses can contribute to more effective care pathways and patient education, while ensuring that technology remains contextualized within each patient’s broader clinical and psychosocial circumstances. The major domains in which artificial intelligence supports obstetrics and gynecology nursing include predictive analytics, intrapartum fetal monitoring, wearable monitoring technologies, and gynecologic oncology screening, as illustrated in Figure 2.

Conceptual framework illustrating the major domains of artificial intelligence applications in obstetrics and gynecology nursing, including predictive analytics, intrapartum monitoring, and gynecologic oncology. The figure demonstrates how these applications support nursing practice through improved risk identification, clinical decision-making, timely intervention, and enhanced patient safety.

Nursing Education and Simulation

Artificial intelligence is increasingly influencing nursing education through adaptive learning platforms, intelligent tutoring systems, and simulation-based training environments. AI-powered virtual patients allow learners to practice managing obstetric emergencies such as shoulder dystocia and postpartum hemorrhage while receiving automated, performance-based feedback [30]. Natural language processing-based chatbots and analytics tools may support the development of clinical reasoning by providing real-time guidance and personalized learning pathways. Performance tracking systems can assist educators in identifying competency gaps and tailoring instruction accordingly.

However, it is important to note that evidence specific to obstetrics and gynecology nursing education remains limited, and much of the existing literature is derived from broader health professions education rather than OBG-focused training contexts. As a result, the applicability of these findings to specialized OBG nursing education requires further investigation. Integrating AI literacy into nursing curricula is increasingly recognized as important to prepare future nurses for technology-enhanced healthcare environments. When combined with traditional experiential learning approaches, AI-based educational tools may enhance learner engagement, confidence, and clinical preparedness; however, further research is needed to evaluate their effectiveness in OBG nursing-specific contexts.

Ethical and Implementation Considerations

Despite its promising applications, AI integration in obstetrics and gynecology nursing raises important ethical, legal, and practical challenges. Algorithmic bias remains a significant concern, particularly when predictive models for conditions such as preeclampsia or gestational diabetes are developed using datasets that underrepresent women from low- and middle-income regions [31]. Such bias may lead to inaccurate risk stratification and delayed recognition of complications in vulnerable populations, thereby perpetuating existing health disparities. Data privacy and security are equally critical, as maternal and reproductive health information, including pregnancy status, genetic screening, and reproductive history, is highly sensitive. The use of AI-enabled monitoring systems and digital platforms necessitates robust data governance frameworks to ensure informed consent, confidentiality, and transparency in how patient data are collected, stored, and used.

Ethical concerns also arise in the use of AI-assisted screening tools, such as cervical cytology or breast cancer detection systems, where unequal access to advanced technologies may widen disparities between high-resource and low-resource settings. Similarly, reliance on automated alerts in maternal early warning systems may risk overdependence on technology if not balanced with clinical judgment. Implementation barriers include high infrastructure costs, limited digital literacy, and inconsistent technological support across healthcare settings [32]. These challenges may particularly affect rural or underserved populations, where access to AI-enhanced care remains limited. Addressing these issues requires interdisciplinary collaboration, strong governance frameworks, and active nurse involvement in AI system design, validation, and policy development. When ethically guided and contextually implemented, AI has the potential to enhance maternal and reproductive healthcare while preserving the human-centered foundation of nursing practice.

Discussion

This narrative review highlights the expanding role of artificial intelligence (AI) in strengthening obstetrics and gynecology (OBG) nursing practice across clinical care, education, and research domains. The findings suggest that AI-driven predictive analytics, clinical decision support systems, wearable monitoring technologies, and oncology applications have the potential to address persistent challenges in maternal and women’s healthcare delivery. However, it is important to recognize that many of these applications remain in developmental, pilot, or early implementation stages, with limited integration into routine clinical practice. While these innovations may be particularly relevant for low- and middle-income countries (LMICs), where shortages of specialist providers and delayed recognition of complications contribute to maternal morbidity and mortality, context-specific evidence on large-scale implementation in such settings remains limited. The following discussion synthesizes the implications of these findings for clinical decision-making, nursing education, ethical governance, and health system readiness.

Enhancing Clinical Decision-Making

AI-based predictive models have demonstrated potential for improved risk stratification for conditions such as preeclampsia, gestational diabetes, and preterm birth when compared with traditional statistical methods [33]. Early identification of high-risk pregnancies may enable nurses to intensify surveillance, reinforce patient education, and coordinate timely multidisciplinary interventions. Similarly, AI-enhanced CTG interpretation and maternal early warning systems may support earlier detection of fetal distress and obstetric emergencies, potentially reducing preventable morbidity. However, it is important to distinguish between proof-of-concept models, externally validated tools, and fully implemented clinical systems, as many AI applications in this domain are still undergoing validation and evaluation. Importantly, AI does not replace clinical reasoning; rather, it augments nursing assessment by synthesizing complex datasets into actionable insights. Adequate training and system familiarity are essential to prevent misinterpretation of automated alerts or excessive reliance on algorithmic outputs. OBG nurses are uniquely positioned to interpret AI-generated insights within the broader clinical and psychosocial context of each patient, ensuring that technology complements rather than replaces individualized, patient-centered care.

Implications for Nursing Education

The integration of AI-based simulation platforms and adaptive learning systems enhances experiential learning in obstetric emergencies. Virtual patient scenarios allow nursing students to practice high-risk but low-frequency clinical events in safe environments while receiving structured feedback [34]. This approach improves critical thinking, clinical preparedness, and decision-making confidence. AI literacy is increasingly becoming a necessary competency for modern nursing professionals. Educational curricula must incorporate foundational knowledge regarding algorithmic functioning, data ethics, and digital health systems to ensure responsible use of technology in practice.

Addressing Ethical and Equity Considerations

Despite promising advancements, AI implementation raises significant ethical concerns. Algorithmic bias remains a critical issue, particularly when models are trained predominantly on datasets from high-income settings, potentially limiting generalizability to diverse populations [35]. In maternal health, such bias could disproportionately affect vulnerable groups. Data privacy and informed consent are equally important, as AI systems rely on large-scale collection and analysis of sensitive reproductive health data. Nurses play a vital advocacy role in safeguarding patient autonomy and ensuring ethical application of digital tools. Transparent governance frameworks, interdisciplinary oversight, and participatory design involving frontline nurses are essential to mitigate unintended consequences.

Health System and Workforce Readiness

Infrastructure limitations, implementation costs, and digital literacy gaps may hinder AI adoption, particularly in low-resource settings [32]. Poorly integrated systems may increase workload through excessive alerts or documentation requirements. Therefore, phased implementation strategies, pilot testing, and capacity-building initiatives are necessary to ensure AI tools enhance rather than burden nursing practice. Future integration of AI with telehealth platforms and digital maternal health records may further expand opportunities for remote monitoring, early risk detection, and continuity of care across pregnancy and postpartum periods. Positioning nurses as collaborators in AI development and evaluation is critical. Their experiential knowledge of workflow, patient interaction, and contextual challenges ensures that technological innovations remain patient-centered and practically feasible.

Limitations

This narrative review has several limitations. As a narrative synthesis, it is inherently susceptible to selection bias and does not employ formal meta-analytic techniques. Although a structured search strategy was followed, the review was not conducted according to formal systematic review guidelines, and a fully reproducible study selection process may not have been achieved. In addition, no formal quality appraisal of included studies was undertaken, which may affect the strength and reliability of the conclusions drawn. The included studies varied considerably in design, outcome measures, validation settings, and stages of implementation, limiting direct comparison.

Furthermore, many AI applications in OBG nursing remain in early developmental or pilot phases, with limited large-scale validation in diverse healthcare systems. Evidence from low- and middle-income countries remains comparatively sparse, which may restrict the generalizability of findings. Continued high-quality, multicenter research, including rigorous validation studies and context-specific implementation research, is required to strengthen the evidence base.

Recommendations

Based on the evidence synthesized in this review, several priority areas for future research and implementation can be identified. First, multicenter validation studies are needed to strengthen the reliability and generalizability of AI-based maternal risk prediction tools, particularly for conditions such as preeclampsia, gestational diabetes, and preterm birth, which were commonly identified in the included studies. Second, there is a need for implementation research evaluating how AI-enabled clinical decision support systems and monitoring technologies can be effectively integrated into nursing workflows, including their impact on clinical decision-making, workload, and patient outcomes. Third, context-sensitive studies in low- and middle-income settings should be prioritized, given the limited representation of such contexts in the current evidence base and the potential relevance of AI in addressing delays in maternal care. In addition, nurse-led innovation and participatory design approaches should be encouraged to ensure that AI tools are aligned with frontline clinical needs and nursing practice realities. Policymakers and healthcare institutions should establish regulatory frameworks that promote ethical deployment, protect patient privacy, and ensure equitable access to digital health technologies. Finally, integrating AI literacy into undergraduate, postgraduate, and continuing professional development programs is essential to prepare OBG nurses for technology-enhanced practice. Structured training initiatives may enhance confidence, support critical appraisal of AI tools, and facilitate their responsible and sustainable integration into clinical care.

## Conclusions

Artificial intelligence is increasingly being explored as a tool to support diagnostic processes, predictive analytics, and clinical decision-making across healthcare. In obstetrics and gynecology nursing, AI-driven technologies show potential to improve maternal and fetal monitoring, facilitate earlier detection of pregnancy-related complications, and support gynecologic cancer screening. These applications may assist healthcare professionals in enabling timely interventions, optimizing patient management, and strengthening healthcare delivery systems. However, current evidence remains heterogeneous and, in many areas, limited to developmental or pilot-stage studies. While the findings are promising, they are not yet sufficient to establish widespread clinical effectiveness across diverse settings. Important methodological, ethical, and implementation challenges, including validation, generalizability, data governance, and equitable access, must be addressed.

The integration of artificial intelligence into nursing education and training also shows potential to enhance clinical competencies and prepare the workforce for technology-enabled care environments, although further research specific to OBG nursing contexts is needed. Overall, artificial intelligence should be viewed as a complementary tool that can support, rather than replace, the clinical judgment and compassionate care provided by nurses. Continued high-quality research, interdisciplinary collaboration, and context-sensitive implementation will be essential to ensure the safe, effective, and equitable integration of AI into obstetrics and gynecology nursing practice.
